# Spatiotemporal analysis of COVID-19 outbreaks in Wuhan, China

**DOI:** 10.1038/s41598-021-93020-2

**Published:** 2021-07-01

**Authors:** Wei Liu, Dongming Wang, Shuiqiong Hua, Cong Xie, Bin Wang, Weihong Qiu, Tao Xu, Zi Ye, Linling Yu, Meng Yang, Yang Xiao, Xiaobing Feng, Tingming Shi, Mingyan Li, Weihong Chen

**Affiliations:** 1 Department of Occupational and Environmental Health, School of Public Health, Tongji Medical College, Huazhong University of Science and Technology, Wuhan, 430030 Hubei China; 2 Key Laboratory of Environment and Health, Ministry of Education and Ministry of Environmental Protection, and State Key Laboratory of Environmental Health (Incubating), School of Public Health, Tongji Medical College, Huazhong University of Science and Technology, Wuhan, 430030 Hubei China; 3 Institute of Preventive Medicine Information, Hubei Provincial Center for Disease Control and Prevention, Wuhan, 430079 Hubei China; 4 Division of Human Resources, Science and Education, Hubei Provincial Center for Disease Control and Prevention, Wuhan, 430079 Hubei China

**Keywords:** Infectious diseases, Respiratory tract diseases

## Abstract

Few study has revealed spatial transmission characteristics of COVID-19 in Wuhan, China. We aimed to analyze the spatiotemporal spread of COVID-19 in Wuhan and its influence factors. Information of 32,682 COVID-19 cases reported through March 18 were extracted from the national infectious disease surveillance system. Geographic information system methods were applied to analysis transmission of COVID-19 and its influence factors in different periods. We found decrease in effective reproduction number (*Rt*) and COVID-19 related indicators through taking a series of effective public health measures including restricting traffic, centralized quarantine and strict stay-at home policy. The distribution of COVID-19 cases number in Wuhan showed obvious global aggregation and local aggregation. In addition, the analysis at streets-level suggested population density and the number of hospitals were associated with COVID-19 cases number. The epidemic situation showed obvious global and local spatial aggregations. High population density with larger number of hospitals may account for the aggregations. The epidemic in Wuhan was under control in a short time after strong quarantine measures and restrictions on movement of residents were implanted.

## Introduction

Coronavirus disease 2019 (COVID-19), a new type of pneumonia caused by Severe Acute Respiratory Syndrome Coronavirus 2 (SARS-CoV-2), was first reported in early December 2019 in Wuhan, China. In mid-January of 2020, some clinicians observed that COVID-19 had strong interpersonal transmission capabilities and could spread through airborne droplets or close contact^1,2^. Subsequently, this disease caused an outbreak in Wuhan. The Chinese government included it as a Class B infectious disease on Jan.20, and required to adopt the prevention and control measures for class A infectious disease which is the strictest control measures.

Wuhan has taken some public health intervention measures to control the spread of COVID-19. On Jan.23, the government required all residents to stay at home as much as possible, interrupted public traffic in the city, and suspended all transport links with other areas. In this period, mild cases and close contacts were required to be isolated at home. After Feb.7, all mild COVID-19 cases were required to be centralized treatment in 14 shelter hospitals. At the same time, suspected cases, cases with fever that cannot be ruled out, and close contacts of confirmed cases were isolation in requisitioning hotels. At the same time, suspected cases, cases with fever that cannot be ruled out, and close contacts of confirmed cases were isolation in requisitioning hotels. On Feb.18, all shops were closed and the residents are required to stay at home. Through these measures, the number of new cases fell fast, below 50 on Mar.8 and no new cases on Mar.18.

Although previous studies predicted or reconstructed the transmission dynamic of COVID-19 in Wuhan and further discussed the impact of non-pharmaceutical interventions based on COVID-19 epidemic temporal changes^3–6^, few study has revealed spatial transmission characteristics. The application of geographic information system (GIS) into routine field epidemiologic surveillance could offer visual evidence for identifying and tracking the spatial spread of infectious diseases^7,8^. In order to learn more from the outbreak in Wuhan, we performed a spatiotemporal analysis of COVID-19 transmission and its potential driving factors in Wuhan as of Mar. 18, 2020 by using GIS methods.

## Materials and methods

### Data source

Data source was well-described in a previous publication^9^. In simple term, information of COVID-19 cases as of March 18 were extracted from the national infectious disease surveillance system, which collected age, sex, residential address (specific to street level), date of illness onset (the self-reported date of symptoms such as fever, cough, or other respiratory symptoms), and date of confirmed diagnosis (the laboratory confirmation date of SARS-CoV-2 in the bio-samples or the date on which the clinician determines the case as a clinically diagnosed case).

The population data (including population size, population density and ratio of the elderly population) was obtained from the statistical yearbooks issued by Wuhan in 2018. The number of public facilities (traffic station, shopping center and hospital) were obtained from Google Maps. Population density was the number of permanent residents per square kilometer; ratio of elderly population was the proportion of the population over 60 years who live permanently in the areas; traffic stations contained both bus stations and subway stations; shopping centers referred to the combinations of retail stores and service facilities in a single building or area that provides comprehensive services to consumers; hospitals with more than 20 beds were included.

### Ethics approval and consent to participate

Data collection and analysis of data were determined by the national infectious disease surveillance system; thus written informed consent or ethics committee/institutional review board approval was not applicable. All subjects were well-informed by the physicians and agreed to report their data to the national infectious disease surveillance system at the time of their medical attention. The system keeps patient information confidential, and all personally identifiable information, such as ID and name, was removed before analyzing the data. Specifically, the addresses of the subjects in this study were only detailed to street level to protect their privacy.

### Case definitions

Diagnosis of confirmed COVID-19 was conducted according to the diagnostic criteria recommended by the National Health Commission of China^10^. Confirmed case was defined as a patient, with corresponding clinical symptoms and a contact history, who had a positive test of SARS-CoV-2 virus by the real-time reverse-transcription-polymerase-chain-reaction (RT-PCR) assay or high-throughput sequencing of nasal and pharyngeal swab specimens.

### Statistical analysis

To better reflect the epidemic of COVID-19, the effective reproduction number (*Rt*) was calculated using the method described by a previous publication^11^. The serial interval (mean: 7.5 days, SD: 3.4 days) derived from a reported of first 425 cases in Wuhan^12^ were applied to estimate *Rt* and its 95% coefficient intervals via a 10-days moving average. According to *Rt* changes at different time, the outbreak was classified into three periods. Period 1: the time before Jan.24, the pre-cognitive period, when no strong intervention was imposed and the epidemic spread naturally. Period 2: Jan. 24–Feb. 7, the control period, the spread of COVID-19 was gradually under control, but the number of cases was still growing (*Rt* more than 1). Period 3: Feb.8-Mar.18, the transmission fading period, (*Rt* less than 1), when all shops were required to close and the residents were required to stay at home. Cumulative cases, average daily new cases, double time and interval from disease onset to diagnosis in different periods were calculated. The doubling time of COVID-19 in each street was calculated according to the equation introduced by Weon^13^. More specific calculation methods of the doubling time and other definitions of COVID-19 indicators were described in the methods section of the supplementary material.

In order to explore the spatial characteristics of COVID-19 spread, we visualized the distribution trend of the onset cases number of each street by constructing a cubic polynomial in different periods on a 3D grid plot. In addition, Moran's *I* was calculated to reflect the global spatial autocorrelation and local spatial autocorrelation of onset COVID-19 cases number distribution in different periods. Monte-Carlo method was used to test the significance of Moran's *I* by simulating 999 times. Cluster map of local indicators of spatial association (LISA) was drawn to show the degree and significance of local cases spatial clustering of one street and its adjacent streets. The modes of local case spatial clustering were divided into five kinds: (1) high–high (area with high cases number surrounded by areas with high cases number), (2) low-low (area with low cases number surrounded by areas with low cases number), (3) low–high (area with low cases number surrounded by areas with high cases number), (4) high-low (area with high cases number surrounded by areas with high cases number), (5) not significant (no significant clustering was found). The calculation method of Moran's *I* was described in detail in a previous literature^14^. In quest of contribution degree of population density and public facilities in each street to COVID-19 onset cases number, Spatial lag model (SLM) was applied to conduct spatial correlation analysis^15^. Given the possibility that the impact of mediators between the possible risk factors and the outcome. We tried to test this possibility with a mediation model (supplementary material).

All analyses were performed with the use of R software (version 3.6.2), ArcGIS 10.2 and GeoDa 1.14.0.0. All figures were created via ArcGIS or GeoDa. All two-sided tests were considered as statistically significant when *P* value was less than 0.05.

### Reporting regulations

Experiments on humans and/or use of human clinical data were not included in this study, so we reported it according to general epidemiological studies.

## Results

### Transmission of COVID-19 in 3 time periods

**Figure 1:**
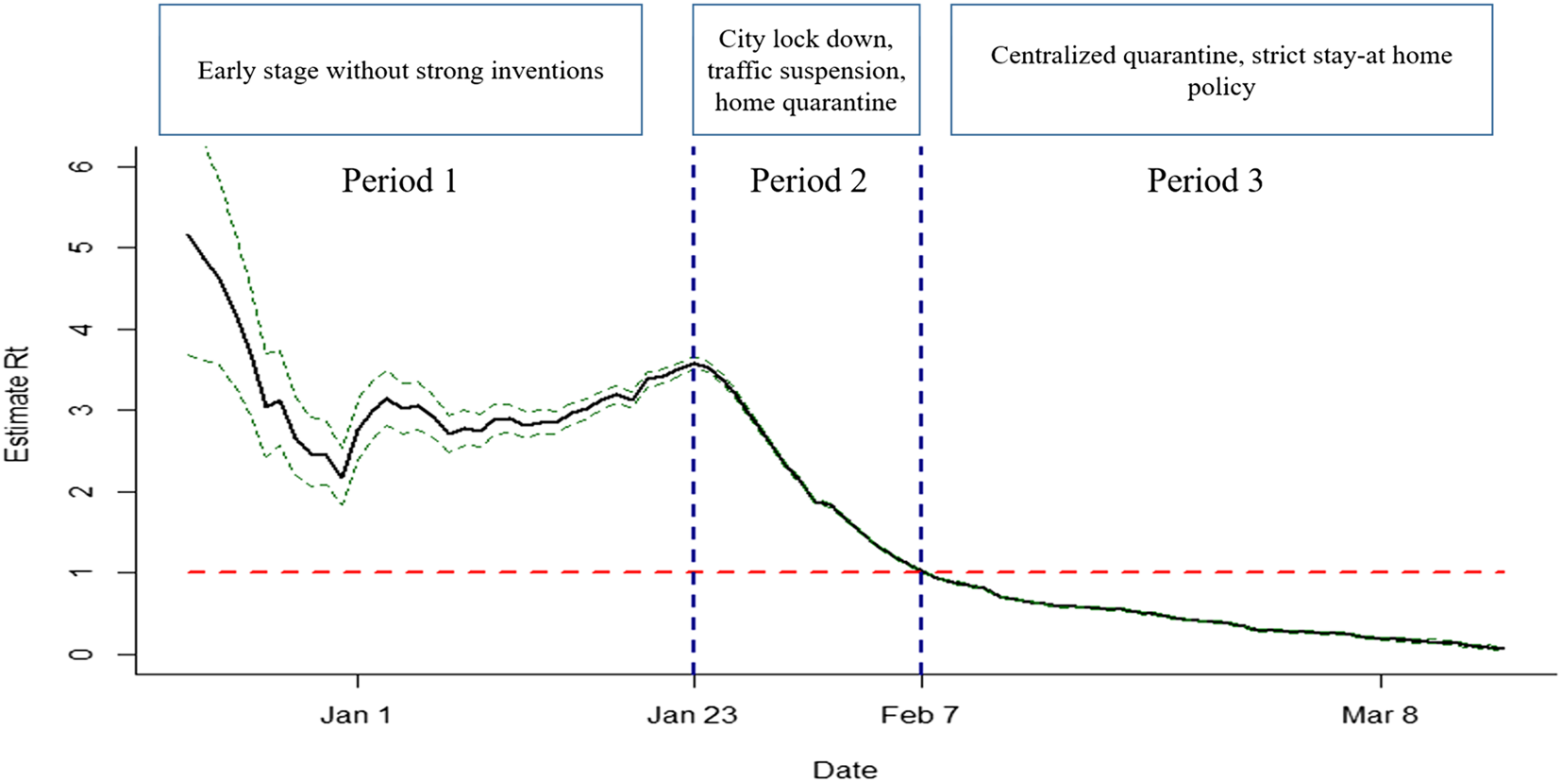
The effective reproduction number (*Rt*) Estimates Based on Coronavirus Disease 2019 (COVID-19) Cases in Wuhan, China. Period 1: the pre-cognitive period, when COVID-19 spread without strong inventions. Period 2: the control period, the spread of COVID-19 is gradually being controlled, but the number of cases is still growing (*Rt* more than 1). Period 3: the transmission fading period (*Rt* less than 1).

By March 18, a total of 32,682 cases were identified from the national infectious disease surveillance system (Table S1). Estimates of the effective reproduction number *Rt* through the whole epidemic period was shown in Fig. 1. The *Rt* varied in the period 1 with a peak of 3.86 on Jan. 23, and declined in the period 2 and 3. The *Rt* fell below 1.0 on Feb. 8, 2020 and further decreased to below 0.1 on Mar. 15, 2020. Basic epidemiological analysis of epidemic differences among different periods was shown in Table 1. The number of onset cases in three periods were 6,981, 18,381 and 7,320, respectively. Average daily new cases in three periods were 166.2, 1,225.4 and 209.1, respectively. Cumulative prevalence (per thousand) raised from 0.6 in period 1 to 2.9 in period 3. Average daily attack rate (per million) in three periods were 0.003, 0.019 and 0.003, respectively. The median of double time elevated from 3.6 days in period 1 to 103.9 days in periods 3, but the median of interval from disease onset to diagnosis decreased form 20.0 day in period 1 to 3.0 days in period 3.

**Table 1:** Transmission of COVID-19 in Wuhan during different period.

Variables	Before Jan. 24, 2020	Jan. 24–Feb. 7, 2020	After Feb. 7, 2020
Onset cases, n	6,981	18,381	7,320
Average daily new cases, n	166.2	1225.4	209.1
Cumulative prevalence, /10^3^	0.6	2.3	2.9
Average daily attack rate, /10^6^	0.003	0.019	0.003
Double time, day	3.6	8.1	103.9
Interval from disease onset to diagnosis, median (IQR), day	20.0 (14.0–25.0)	11.0 (8.0–16.0)	3.0 (1.0–5.0)

COVID-19, coronavirus disease 2019; IQR, interquartile range.

### The spatiotemporal distribution of COVID-19 cases in Wuhan

**Figure 2:**
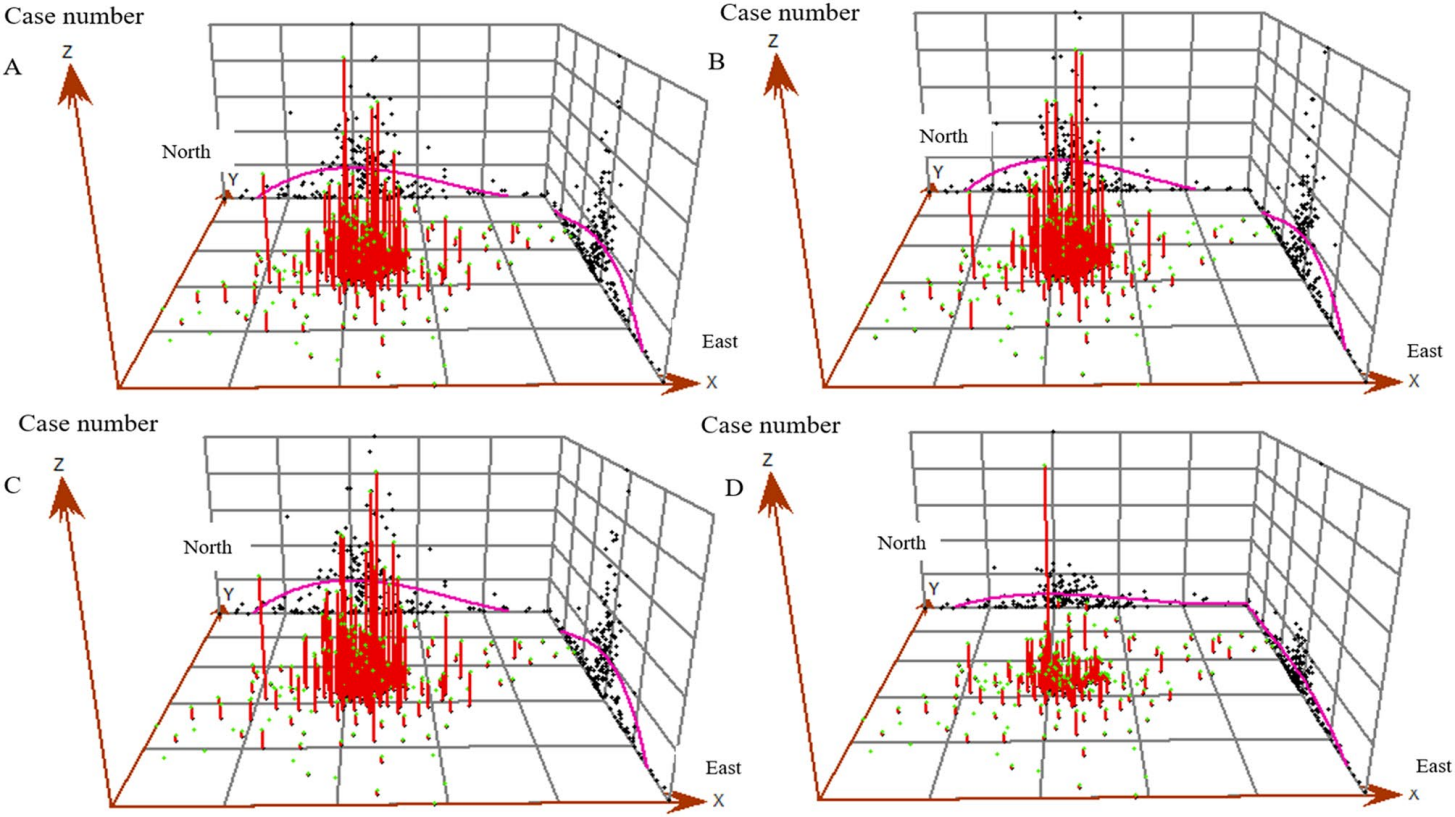
Street-level global spatial trend of onset COVID-19 cases Wuhan, China in different periods, respectively. (**A**) The whole epidemic time (from Dec. 8, 2019 to Mar. 18, 2020). (**B**) Period 1, the pre-cognitive period, when COVID-19 spread without strong inventions. (**C**) Period 2, the control period, the spread of COVID-19 is gradually being controlled, but the number of cases is still growing (*Rt* more than 1). (**D**) Period 3, the transmission fading period (*Rt* less than 1). The X-axis points to the north of Wuhan, the Y-axis points to the east of Wuhan, and the Z-axis is cases number. The points on the grid are projections of cases number in each street. The curve on the grid shows the distribution trend of cases in overall city. The red column represents the cases number in each street.

**Figure 3:**
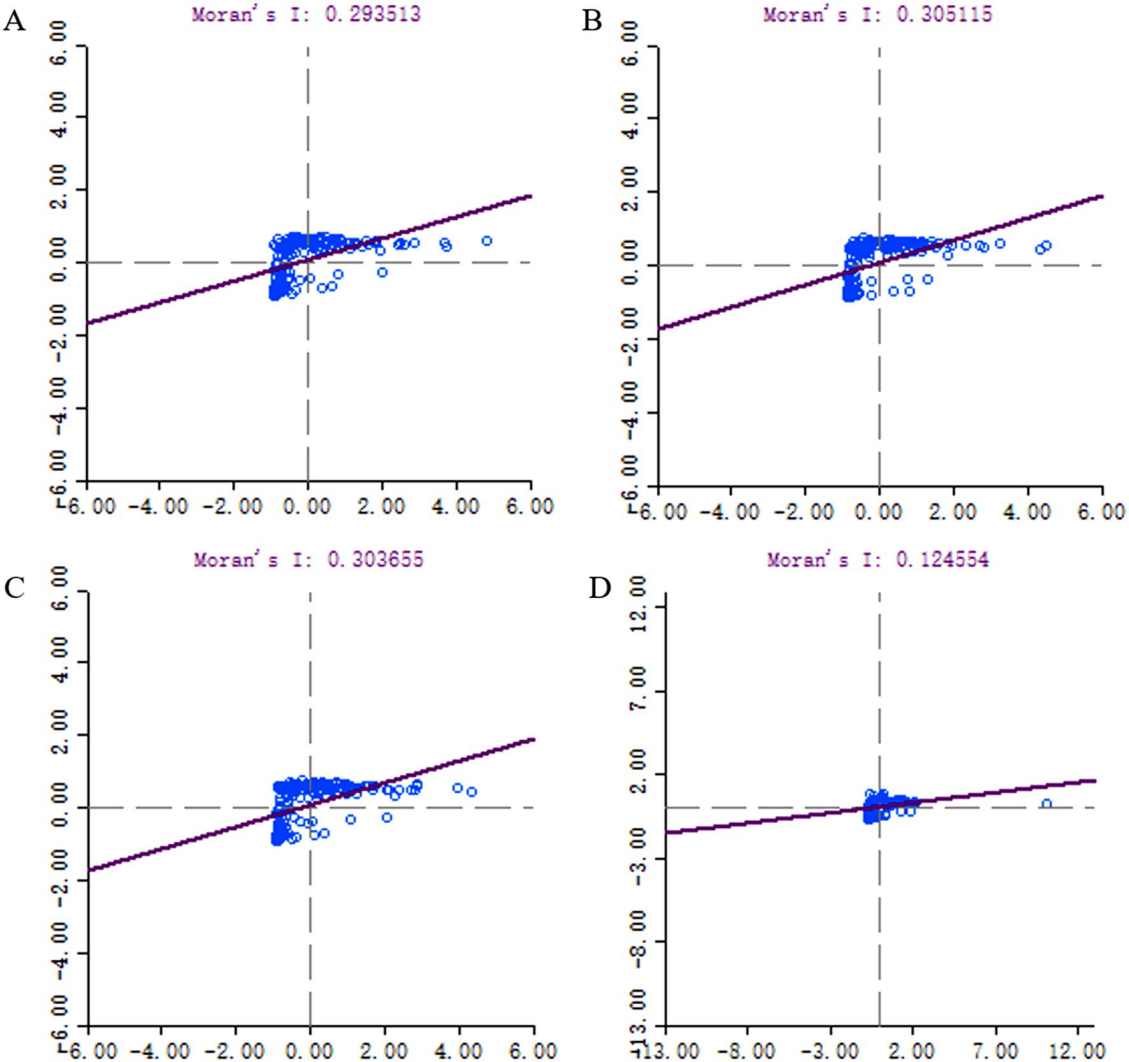
Moran scatter plot of onset COVID-19 cases spatial autocorrelation in streets of Wuhan city. The X-axis is the standardized value of cases number, and the Y-axis is the standardized value of the cases number in adjacent streets. The bubbles represent all streets of Wuhan city. (**A**) The whole epidemic time (from Dec. 8, 2019 to Mar. 18, 2020). (**B**) Period 1, the pre-cognitive period, when COVID-19 spread without strong inventions. (**C**) Period 2, the control period, the spread of COVID-19 is gradually being controlled, but the number of cases is still growing (*Rt* more than 1). (**D**) Period 3, the transmission fading period (*Rt* less than 1).

A total of 179 streets in Wuhan city were included in the present analysis and COVID-19 cases were reported from 177 of them. Global spatial trends in whole epidemic and 3 time periods were visualized in Fig. 2. The trend lines suggested COVID-19 cases aggregated in central urban area in all periods, but such overall trend of aggregation reduced obviously in period 3. Global spatial autocorrelations in whole epidemic and different periods were examined by Moran's *I* (Fig. 3). In all Moran scatter plots, bubbles mainly aggregated in the first, second and third quadrants, suggested that the spatial distribution form of COVID-19 onset cases in all period were mainly composed of three main patterns: high–high, low–high and low–low. Moran's *I* in all periods was more than 0, but decreased from 0.31 in period 1 to 0.12 in period 3. Significance tests of Moran's *I* performed by Monte-Carlo method with 999-time simulations indicated significant (pseudo p value < 0.05) global autocorrelation existed in all periods (Figure S1).

**Figure 4:**
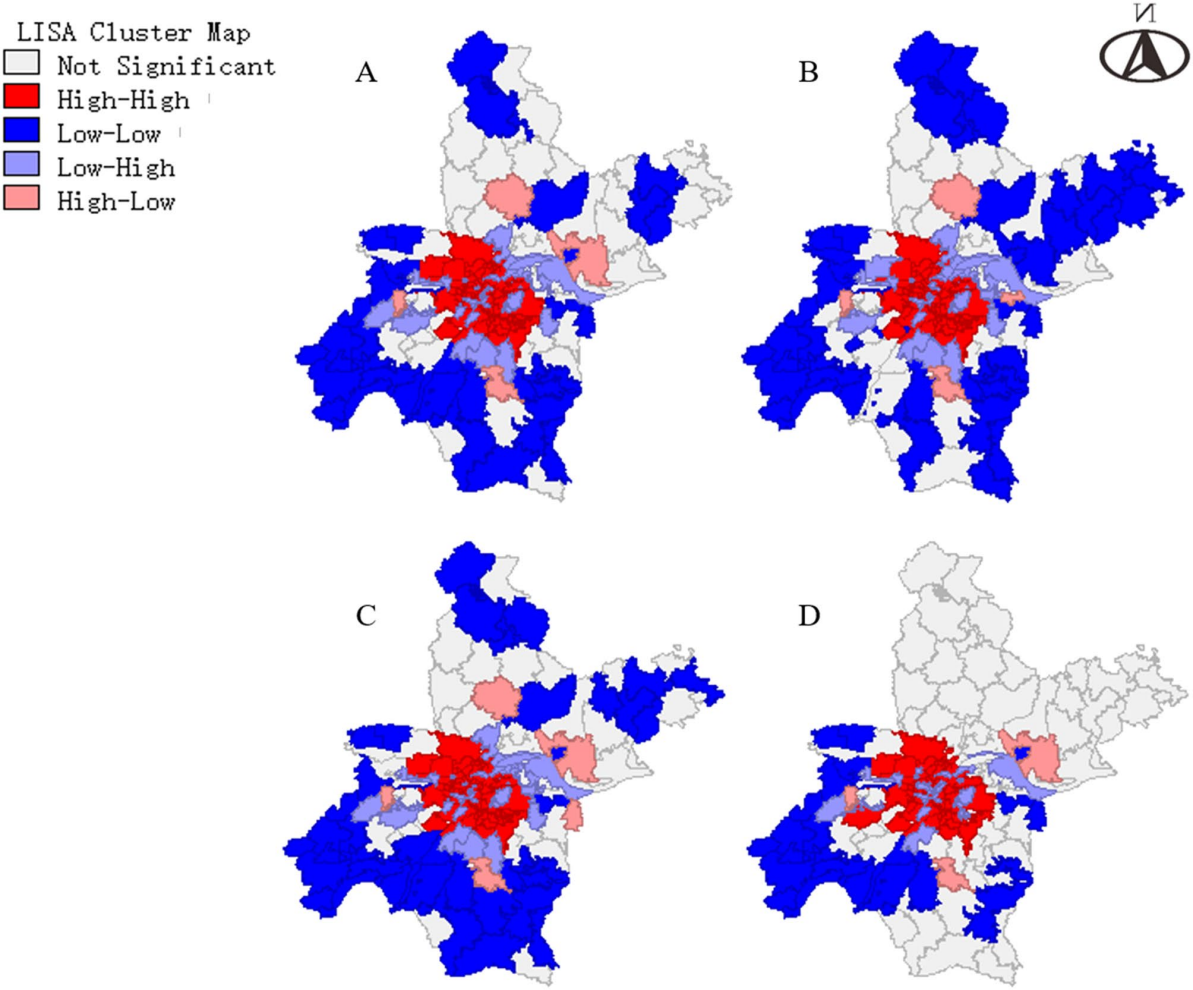
Lisa cluster map of onset COVID-19 cases local spatial autocorrelation of Wuhan city in street-level. (**A**) The whole epidemic time (from Dec. 8, 2019 to Mar. 18, 2020). (**B**) Period 1, the pre-cognitive period, when COVID-19 spread without strong inventions. (**C**) Period 2, the control period, the spread of COVID-19 is gradually being controlled, but the number of cases is still growing (*Rt* more than 1). (**D**): Period 3, the transmission fading period (*Rt* less than 1). The map was created via software GeoDa (1.14.0.0, URL http://geodacenter.github.io/download.html). The map data was obtained from a public website (https://data.wuhan.gov.cn/page/data/data_set_details.html?cataId=72a1127f-ffa1-11ea-8202-00ff97c29d31).

In order to have a more detailed view of spatial distribution of COVID-19 onset cases in different periods, LISA cluster map was employed to graphically demonstrate local autocorrelation of COVID-19 onset cases in street-level (Fig. 4). From the perspective of the whole epidemic, the main models of onset cases clustering from the central urban area to the marginal urban area were high–high, high–low or low–high, and low-low, successively. As shown in Table 2, the number of streets which did not present significant clustering elevated from 18 in period 1 to 54 in period 3. Closer inspection of the Table 2 showed such trend of reduction was due to the decrease in high–high and low-low aggregation.

**Table 2:** Street-level spatial clustering models of COVID-19 onset cases in different periods.

Period	Number of different spatial clustering models				
	High–high	High–low	Low–high	Low–low	Not significant
The whole epidemic	68	4	43	37	27
Period 1	69	4	41	47	18
Period 2	70	5	43	39	22
Period 3	59	3	39	24	54

Significance of local spatial clustering was tested by local Moran's I. Period 1, the pre-cognitive period, when COVID-19 spread without strong inventions. Period 2, the control period, the spread of COVID-19 is gradually being controlled, but the number of cases is still growing (*Rt* more than 1). Period 3, the transmission fading period (*Rt* less than 1).

### Analysis of spatial differentiation drivers

To explore the driving factors of COVID-19 cases spatial differentiation, we performed a tertile analysis of the street according to the population density or the number of public facilities in each street (Table S2). The results suggested that all COVID-19 indictors (including cumulative number of case, average prevalence, doubling time and daily new cases were monotonic increase across tertiles of population density (all *P*_trend_ < 0.05). The number of daily new cases in three periods, as well as the average prevalence and the cumulative cases of COVID-19 (all *P*_trend_ < 0.05) elevated significantly with the increase in the number of hospitals. We didn’t observe any one-way variation trend between shopping center (except number of average daily new cases) and other COVID-19 related indicators, or between the number of traffic station and COVID-19 indicators.

To further validate such potential associations, spatial lag models were constructed to detect the association of the number of COVID-19 onset cases with population density, ratio of the elderly population and number of public facilities in street-level. As shown in Table 3, population density (coefficient: 0.001) and number of hospitals (coefficient: 27.236) were significantly associated with the number of onset cases at street-level (both *P* < 0.05) rather than ratio of elderly population and the number of other public facilities throughout the whole epidemic. When stratified into three periods, significant associations of onset cases with population density (coefficient: 0.001 in period 1 and 2) and the number of hospitals (coefficient: 5.660 in period 1, 14.694 in period 2) were observed in period 1 and 2. In addition, the number of traffic stations was positively associated with onset cases with a coefficient of 4.416 in period 2. Strikingly, no significant association between population density and onset cases was found in period 3. Nonetheless, the number of hospitals was still positive associated with onset cases elevation in period 3, but the coefficient was lower than that in period 2 (6.928 vs 14.694). In further mediation analysis significant mediation effect of number of hospitals on the association between population density and COVID-19 cases number of whole epidemic was observed. The mediation proportion was 29.7% (Figure S2).

**Table 3:** Street-level correlation of COVID-19 cases number with population density and the number of public facilities of Wuhan city in different periods.

Periods	Characteristics	Coefficient	Std. Error	z-value	*P* value for coefficient
The whole epidemic	Population density	0.001	0.001	3.574	< 0.001
	Ratio of the elderly populations	0.537	3.073	0.175	0.861
	Number of traffic stations	6.321	3.543	1.784	0.074
	Number of shopping centers	− 5.994	4.796	− 1.249	0.211
	Number of hospitals	27.263	7.035	3.875	< 0.001
Period 1	Population density	0.001	0.001	3.142	< 0.001
	Ratio of the elderly populations	− 0.005	0.703	− 0.007	0.994
	Number of traffic stations	1.578	0.813	1.942	0.052
	Number of shopping centers	− 1.628	1.100	− 1.480	0.139
	Number of hospitals	5.660	1.613	3.508	< 0.001
Period 2	Population density	0.001	0.001	2.989	< 0.001
	Ratio of the elderly populations	− 0.498	1.725	− 0.289	0.773
	Number of traffic stations	4.416	1.992	2.217	0.027
	Number of shopping centers	− 3.941	2.697	− 1.461	0.144
	Number of hospitals	14.694	3.956	3.715	< 0.001
Period 3	Population density	0.001	0.001	0.098	0.151
	Ratio of the elderly populations	1.260	0.952	1.324	0.186
	Number of traffic stations	0.377	1.098	0.343	0.731
	Number of shopping centers	− 0.507	1.486	− 0.341	0.733
	Number of hospitals	6.928	2.180	3.176	< 0.001

Spatial lag model was applied to detect the correlation of COVID-19 cases number with population density and the number of public facilities. Period 1, the pre-cognitive period, when COVID-19 spread without strong inventions. Period 2, the control period, the spread of COVID-19 is gradually being controlled, but the number of cases is still growing (*Rt* more than 1). Period 3, the transmission fading period (*Rt* less than 1).

## Discussion

The present study found that the transmission of COVID-19 in Wuhan experienced three periods of outbreak, control and decline in time, and presented spatial clustering in the central urban area. In addition, population density and the number of hospitals were both positive associated with COVID-19 indicators at streets-level.

In the early stage, the *Rt* reached a peak on Jan.23. However, the government intervened with a series of public health measures after the discovery of conclusive evidence that COVID-19 could be passed from person to person^16^. The present study divided the epidemic of COVID-19 in Wuhan into three periods. In period 1, when no strong intervention was implemented, the doubling time of COVID-19 cases was 3.6 days, which was shorter than the 7.5 (5.3–19) days calculated by model simulations in an earlier study^12^. Such difference may be due to the limitation of detection capacity in the early stage of the outbreak, resulting some cases not being confirmed in a timely manner and the transmission not being properly assessed. In period 2, indicators of transmission, including onset cases and average daily new cases indicated that the epidemic was still rising, but changes in doubling time and *Rt* both suggested the epidemic was under control in some degree. On one hand, as the median incubation period of COVID-19 is up to 14 days^17,18^, changes in indicators may lag behind the impact of intervention measures. On the other hand, mild and suspected cases were required to isolate at home in that period, which still had a great risk of transmission, especially in areas with high population density. In period 3, the doubling time increased more than 10 times that of the previous period. In fact, almost all of the identified potential infectors were isolated in the period 3, and the strict stay-at-home policy for all residences cut off transmission to a great extent. Therefore, strict measures to isolate and limit population movements, rather than just restricting public transportation and population gathering, are needed to control the outbreak of COVID-19 in a short time.

The present study found that the epidemic situation showed obvious aggregation in central urban areas, where found the first case. In three periods, significant spatial autocorrelations of COVID-19 onset cases number in Wuhan were found, especially in period 1 and 2. The transmission of COVID-19 in first two periods tended to spread from high-incidence areas to low-incidence areas. The size of aggregation reduced in the later stage (after the implementation of strict population movement control measures, period 3) of the epidemic. Such a change in spatial distribution characteristics suggested that the maximum restriction of human movement during the outbreak may have a significant effect, especially in high-incidence areas.

Our study also found that the population density as well as the number of hospitals in the streets was associated with COVID-19 indicators. In addition, the number of hospital may act an important mediation role. Studies have proposed that hospital may become a source of infection due to public health emergency^19^. Several studies^20,21^ on the investigation of nosocomial infection concluded that the incidence of COVID-19 due to the nosocomial infection is not low. An investigation of 662 inpatients with COVID-19 at an NHS Trust in South London suggested that 45 (6.8%) inpatients were likely infected while seeking medical attention^20^. An analysis of 138 COVID-19 cases conducted by a hospital in Wuhan showed that the ratio of nosocomial infection was up to 41.3%^21^. In fact, large number of residences with similar or suspected symptoms of COVID-19 flocked to hospitals to seek for treatment, which not only led to the directional movement of cases, but also increased the risk of cross-infections. However, a number of public health interventions were implemented by the Wuhan government from Jan.23 to Feb.18, including shutdown of public gathering places, restrictions of inner-city traffic, and strict stay-at-home policy for all residences. These effective interventions might lead to the fact we did not observe the association of traffic stations with increased number of average daily new cases. Restricting traffic eliminated the impact of the number of stations on COVID-19 indictors. It is surprising that no association was observed between ratio of elderly population and the number of onset cases, even though multiple studies^3–6^ and our results jointly confirmed the susceptibility of elderly to COVID-19. We thought that it may be because area with ratio of elderly population had lower population density and some of them are located in remote areas^22^. The lower population density and lower population mobility resulted in the reduced a lower probability of infection among the residents in these areas.

Application of GIS methods in infectious diseases were may provide additional epidemiological clues for COVID-19 outbreak. For example, Rui Huang et al.^23^ made a prediction on spatial–temporal distribution of COVID-19 in China at the early stage of the epidemic by constructing GIS model. In addition, Mohsen Shariati et al.^24^ used hot spot analysis coupled with Anselin local Moran's *I* to determine the high-risk districts of COVID-19 over the world. The present study performed a spatiotemporal analysis of the COVID-19 transmission in Wuhan, China for the first time. Further investigations are needed to identify more spatial characteristics of COVID-19 epidemic. This is of important public health implications, especially in terms of providing a basis for public health measure.

There are some limitations in this study. First, the retrospective observational study design precludes causal inference. Second, due to date were extracted from the national infectious disease surveillance system, other extraneous factors, such as incubation period, medical treatment strategies, and vital status was not available. Therefore, counterfactual control may not be enough. Third, street characteristics data and COVID-19 cases data were not from the same data source. This may lead to the possibility of bias in the results.

## Conclusion

The epidemic of COVID-19 in Wuhan shows obvious aggregation. High population density and high number of hospitals may be risk factors for the transmission of the COVID-19 in Wuhan. The spatiotemporal analysis of COVID-19 transmission in Wuhan suggest that maximum restriction of human movement and strict isolation should be taken into consideration in order to control the outbreak in a short time.

## Supplementary Information

Supplementary Information.

## Data Availability

The datasets used and/or analyzed in the current study are available from the corresponding author on reasonable request. Contact information for the data access committee: hbcdc_limingyan@163.com (e-mail).
