# Supplementary material for: Spatiotemporal analysis of COVID-19 outbreaks in Wuhan, China
Source: Sci Rep. 2021 Jul 1;11:13648. doi: 10.1038/s41598-021-93020-2 (PMC8249501; doi:10.1038/s41598-021-93020-2)
Supplement: Supplementary file 1 — Supplementary Information. [file 41598_2021_93020_MOESM1_ESM.docx]

Supplementary material

**Spatiotemporal analysis of COVID-19 outbreaks in Wuhan, China**

Wei Liu, MS ^1,2,#^, Dongming Wang, MD ^1,2,#^, Shuiqiong Hua, MD ^3,#^, Cong Xie, MS ^3,#^, Bin Wang, MD ^1,2^, Weihong Qiu, MD ^1,2^, Tao Xu, MS ^1,2^, Zi Ye, MS ^1,2^, Linling Yu, MS ^1,2^, Meng Yang, MD ^1,2^, Yang Xiao, MS ^1,2^, Xiaobing Feng, MS ^1,2^, Tingming Shi, MD ^4^, Mingyan Li, MD ^3*^, Weihong Chen, MD ^1,2*^

**Affiliations:**

^1^ Department of Occupational & Environmental Health, School of Public Health, Tongji Medical College, Huazhong University of Science and Technology, Wuhan, Hubei 430030, China

^2^ Key Laboratory of Environment and Health, Ministry of Education & Ministry of Environmental Protection, and State Key Laboratory of Environmental Health (Incubating), School of Public Health, Tongji Medical College, Huazhong University of Science and Technology, Wuhan, Hubei 430030, China

^3^ Institute of Preventive Medicine Information, Hubei Provincial Center for Disease Control and Prevention, Wuhan, Hubei 430079, China

^4^ Division of Human Resources, Science and Education, Hubei Provincial Center for Disease Control and Prevention, Wuhan, Hubei 430079, China

# These authors contributed equally to this work

***Corresponding author:**

Prof. Weihong Chen

Department of Occupational and Environmental Health, School of Public Health, Tongji Medical College, Huazhong University of Science and Technology, Wuhan, Hubei 430030, China

Tel: +86 27 83691677; fax: +86 27 83691677

E-mail: [wchen@mails.tjmu.edu.cn](mailto:wchen@mails.tjmu.edu.cn)

***Co-corresponding author:**

Dr. Mingyan Li

Institute of Preventive Medicine Information, Hubei Provincial Center for Disease Control and Prevention Hubei 430079, China

Tel: +86 027-87652021

E-mail: [hbcdc_limingyan@163.com](mailto:hbcdc_limingyan@163.com)

**Supplementary methods**

To assess the mediating role of number of hospitals in the association between urinary population density and COVID-19 cases, we employed an R package MEDIATION to perform mediation analysis which has been described previously [1]. In short, two general line models (Equation (1) and (2)) were fitted to estimated direct effect and indirect effect.

*M = θ_0_ + β_population density_X_population density_ + β_c_X_c_ + λ_1_*  (1)

*Y = θ_1_ + μ_population density_X_population density_ + μ_M_X_M_ + β_c_X_c_ + λ_2_*  (2)

Where, *Y* signifies outcome (COVID-19 cases), *X_population density_* signifies exposure (population density), *M* signifies mediator (number of hospitals), *X_c_* signifies confounders. Direct effect equals to *μ_population density_*, and the product of *β_population density_* and *μ_M_* denotes the mediated effect. Proportion mediated was calculated as *β_population density_*×*μ_M_* /(*β_population density_*×*μ_M_* +*μ_2HPM_*)×100%.

On account of a series of measures imposed by the government to limit people movement (including city lockdown, traffic suspension and strict stay-at-home policy), the movement of residents in Wuhan city was largely confined to the streets where they lived. Therefore, we explored variation of population density and public facilities in each street to COVID-19 elated indicators.

We calculated the prevalence and daily attack rate of each street, which were defined as case number divided by the number of street residents (per 10^3^ people), and the number of daily new cases divided by the number of street residents (per day per 10^3^ people), respectively. In order to facilitate comparison, we assume that the increase of COVID-19 cases in Wuhan has been exponential all the time. Therefore, the doubling time of COVID-19 in each street was calculated according to the equation introduced by Weon [2]:

*α (t) = Δt × ln 2 / ln (Nt / N_0_)*

Here, *α* (*t*) is the doubling time; *Nt* is the cumulative number of cases on the last day of each period; *N_0_* is the cumulative number of cases on the first day of each period, but if the cumulative cases of a street on the first day of this period were zero, we set *N_0_* as 1; *Δt* is the days which cost for the cumulative number of cases to increase from *N_0_* to *Nt*.

According to the tertiles of the population density or the number of different types of public facilities, streets were divided into three categories: low, moderate, and high. Variance analysis was used to the distribution trend of COVID-19 related indicators across tertiles of the population density, ratio of elderly population or the number of different types of public facilities (with low category as a reference).

Reference

1. Tingley D, Yamamoto T, Hirose K, Keele L, Imai K (2013). mediation: R Package for Causal Mediation Analysis. R package version 4.4.2, URL http://CRAN.R-project.org/ package=mediation
2. Weon BM. Doubling time tells how effective Covid-19 prevention works. medRxiv. 2020:2020.03.26.20044644. doi:10.1101/2020.03.26.20044644

**Supplementary Results**

**Table S1.** Epidemiological characteristics and key time-to-event distributions of total cases

| Variables | No. (%) |
| --- | --- |
|  | Total cases |
| Total | 32,682 |
| Age group, years |  |
| <20 | 537 (1.6) |
| 20- | 6,023 (18.4) |
| 40- | 12,273 (37.6) |
| 60- | 11,951 (36.6) |
| 80- | 1,898 (5.8) |
| Sex |  |
| Male | 15,814 (48.4) |
| Female | 16,868 (51.6) |
| Clinical classification^a^ |  |
| Mild | 15,535 (47.5) |
| Moderate | 9,729 (29.8) |
| Severe | 61,164 (18.9) |
| Critical | 1,009 (3.1) |
| Symptom onset period^b^ |  |
| Dec.8-17, 2019 | 14 (0.0) |
| Dec.18-27, 2019 | 56 (0.2) |
| Dec.28, 2019-Jan.6, 2020 | 261 (0.8) |
| Jan.7-16, 2020 | 1,509 (4.6) |
| Jan.17-26, 2020 | 9,273 (28.4) |
| Jan.27-Feb.5, 2020 | 12,809 (39.2) |
| Feb.6-15, 2020 | 5,331 (16.3) |
| Feb.16-25, 2020 | 2,879 (8.8) |
| After Feb.25, 2020 | 553 (1.7) |
| Diagnosis period |  |
| Before Jan.24, 2020 | 401 (1.2) |
| Jan.24-Feb.2, 2020 | 4,417 (13.5) |
| Feb.3-12, 2020 | 15,542 (47.6) |
| Feb.13-22, 2020 | 8,339 (25.5) |
| Feb.23-Mar.3, 2020 | 3,504 (10.7) |
| After Mar.3, 2020 | 479 (1.5) |
| Interval from disease onset to diagnosis, median (IQR), day | 11 (6,17) |

Abbreviations: COVID-19, coronavirus disease 2019; IQR, interquartile range.

^a^ A total of 245 cases were reported with miss information.

^b^ One case was reported with miss information.


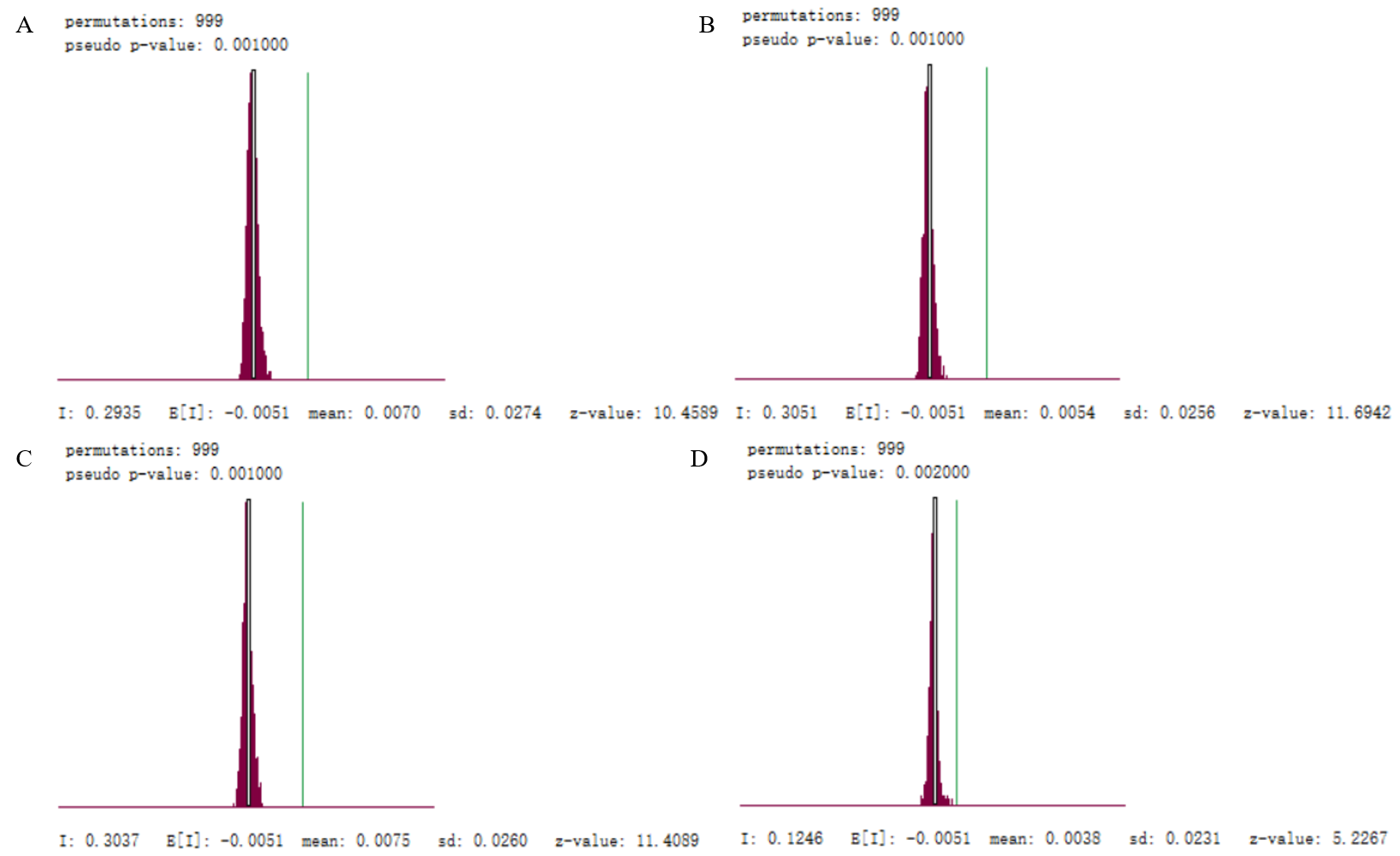


**Figure S1** **Monte-Carlo test chart of Moran's *I* of onset COVID-19 cases number spatial autocorrelation in streets of Wuhan city.** A: The whole epidemic time (from Dec. 8, 2019 to Mar. 18, 2020). B: Period 1, the pre-cognitive period, when COVID-19 spread without strong inventions. C: Period 2, the control period, the spread of COVID-19 is gradually being controlled, but the number of cases is still growing (*Rt* more than 1). D: Period 3, the transmission fading period (*Rt* less than 1). Monte-Carlo method was used to test the normality of Moran's *I* by simulating 999 time.


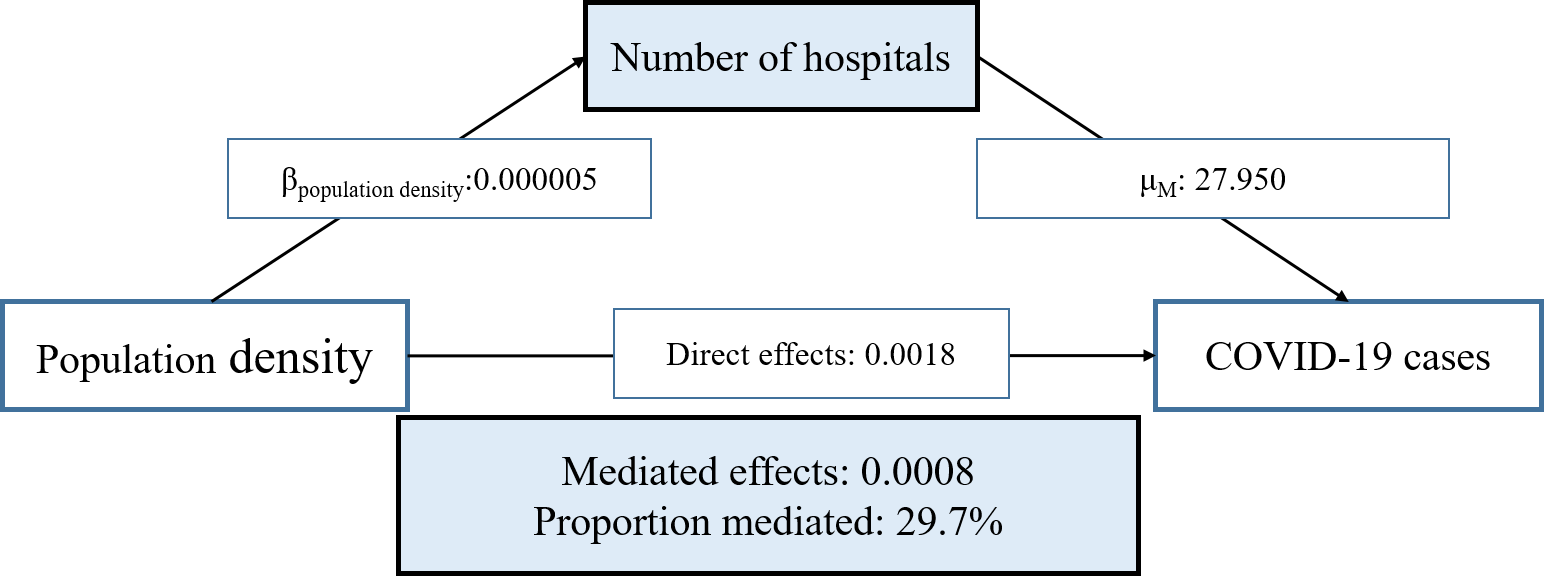


**Figure 2. Assessment of mediating effect of hospitals number on the associations between population density and COVID-19 cases number of whole epidemic.** All models were adjusted for number of traffic station, number of shopping center, number of hospital and ratio of elderly population.

**Table S2. Association of street population density and public facilities with COVID-19 by taking streets as basic units**

|  | **Population density, / km²** | | |  | **Number of traffic station, N** | | |  | **Number of shopping center, N** | | |  | **Number of hospital, N** | | |  | **Population density, / km²** | | | |
| --- | --- | --- | --- | --- | --- | --- | --- | --- | --- | --- | --- | --- | --- | --- | --- | --- | --- | --- | --- | --- |
| **Variables** | **Low (≤544.92)** | **Moderate (544.92-11851.85)** | **High (>11851.85)** | ***P*_trend_^a^** | **Low (≤2)** | **Moderate (3-6)** | **High (≥7)** | ***P*_trend_^a^** | **Low (0)** | **Moderate (1-2)** | **High (≥3)** | ***P*_trend_^a^** | **Low (≤1)** | **Moderate (2)** | **High (≥3)** | ***P*_trend_^a^** | **Low (≤19.8%)** | **Moderate (19.8-24.3)** | **High (>24.3)** | ***P*_trend_^a^** |
| **Number of street, N** | 60 | 59 | 60 |  | 60 | 70 | 49 |  | 79 | 53 | 47 |  | 104 | 34 | 41 |  | 55 | 57 | 54 |  |
| **Cumulative cases median (IQR), N** | 27 (9.5-43) | 147.0 (69.5-283.0) | 271.0 (191.8-405.3) | <0.001 | 77.0 (22.5-283.5) | 103.0 (32.0-256.0) | 183.5 (86.2-318.8) | 0.108 | 91.0 (23.0-238.5) | 147.0 (42.5-352.5) | 114.5 (62.5-240.0) | 0.114 | 56.0 (23.5-183.0) | 137.5(67.3-243.5) | 277.0 (197.0-423.5) | <0.001 | 67.0 (16.5-229.5) | 56.0 (31.0-198.0) | 199.0 (112.5-334.3) | 0.252 |
| **Daily new cases, median (IQR), N** |  |  |  |  |  |  |  |  |  |  |  |  |  |  |  |  |  |  |  |  |
| Period 1 | 0.1 (0.0-0.2) | 0.6 (0.2-1.4) | 1.3 (1.0-1.8) | <0.001 | 0.2 (0.0-1.3) | 0.5 (0.1-1.2) | 1.0 (0.2-1.6) | 0.196 | 0.4 (0.0-1.2) | 1.0 (0.2-1.7) | 0.6 (0.2-1.1) | 0.262 | 0.2 (0.0-0.7) | 0.5 (0.2-1.2) | 1.3 (1.0-1.8) | <0.001 | 0.2 (0.1-1.1) | 0.2 (0.1-0.9) | 1.0 (0.5-1.4) | 0.196 |
| Period 2 | 0.9 (0.3-1.3) | 6.1 (2.2-9.9) | 10.3 (6.9-25.1) | <0.001 | 2.8 (0.7-10.4) | 3.9 (1.0-9.3) | 7.0 (3.0-10.7) | 0.113 | 3.7 (0.9-9.0) | 6.1 (1.1-14.6) | 4.6 (1.9-9.4) | 0.115 | 1.9 (0.8-7.1) | 5.8 (2.2-9.8) | 9.7 (7.5-17.2) | <0.001 | 1.9 (0.7-8.9) | 1.9 (0.9-7.4) | 7.5 (4.5-13.1) | 0.211 |
| Period 3 | 0.2 (01.-0.5) | 0.8 (0.5-1.4) | 1.3 (0.8-1.9) | <0.001 | 0.5 (0.2-1.2) | 0.7 (0.3-1.2) | 0.8 (0.5-1.6) | 0.23 | 0.6 (0.2-1.2) | 0.8 (0.4-1.6) | 0.7 (0.5-1.2) | 0.889 | 0.5 (0.2-1.0) | 0.8 (0.5-1.5) | 1.5 (0.9-1.5) | 0.002 | 0.5 (0.1-1.0) | 0.7 (0.3-1.2) | 0.9 (0.5-1.8) | 0.051 |
| **Average prevalence, per 10^3^ people** | 0.8 | 3.1 | 3.6 | <0.001 | 2.6 | 2.4 | 2.6 | 0.825 | 2.4 | 2.2 | 3 | 0.388 | 1.8 | 3.2 | 3.4 | 0.007 | 1.1 | 3.5 | 2.4 | 0.458 |
| **Double time, median (IQR), day** |  |  |  |  |  |  |  |  |  |  |  |  |  |  |  |  |  |  |  |  |
| Period 1 | 3.8 (2.5-6.0) | 4.1 (3.1-5.0) | 3.8 (2.9-4.8) | 0.107 | 3.6 (2.8-4.8) | 3.7 (2.8-4.8) | 4.6 (3.4-6.2) | 0.071 | 3.8 (2.8-4.8) | 3.8 (3.0-4.8) | 4.2 (3.1-3.2) | 0.624 | 3.8 (2.9-4.8) | 3.8 (2.9-5.0) | 4.1 (3.3-6.1) | 0.786 | 4.2 (2.6-6.3) | 4.4 (2.1-7.0) | 4.6 (3.1-5.8) | 0.587 |
| Period 2 | 5.6 (4.0-7.3) | 7.0 (6.3-7.9) | 7.4 (6.5-7.9) | 0.972 | 7.0 (5.7-8.6) | 6.8 (5.6-7.7) | 6.5 (5.7-7.7) | 0.302 | 6.6 (5.4-7.7) | 7.0 (5.9-8.0) | 6.8 (5.8-7.6) | 0.671 | 6.6 (4.9-7.8) | 6.8 (5.8-7.6) | 7.5 (6.5-8.0) | 0.957 | 8.0 (5.2-1.0.0) | 8.2 (5.6-9.9) | 8.6 (7.6-11.0) | 0.777 |
| Period 3 | 51.2 (36.6-65.4) | 76.5 (57.0-109.2) | 103.0 (74.1-117.7) | <0.001 | 74.2 (42.4-115.0) | 65.2 (46.8-105.3) | 73.1 (59.0-101.6) | 0.891 | 73.0 (52.2-103.3) | 78.4 (57.4-78.4) | 70.6 (46.4-106.3) | 0.461 | 65.2 (44.4-104.2) | 67.4 (55.2-97.3) | 94.1 (69.6-115.5) | 0.262 | 121.4 (74.6-161.8) | 65.3 (42.0-107.2) | 132.7 (103.3-165.3) | 0.481 |

Abbreviations: COVID-19, coronavirus disease 2019; IQR, interquartile range. Period 1, the pre-cognitive period, when COVID-19 spread without strong inventions. Period 2, the control period, the spread of COVID-19 is gradually being controlled, but the number of cases is still growing (*Rt* more than 1). Period 3, the transmission fading period (*Rt* less than 1).

^a^ *P*_trend_ were estimated by variance analysis with adjusting for population density, ratio of elderly population and number of public facilities.
